# Enhanced Deep Learning Approach for Accurate Eczema and Psoriasis Skin Detection

**DOI:** 10.3390/s23167295

**Published:** 2023-08-21

**Authors:** Mohamed Hammad, Paweł Pławiak, Mohammed ElAffendi, Ahmed A. Abd El-Latif, Asmaa A. Abdel Latif

**Affiliations:** 1 EIAS Data Science Lab, College of Computer and Information Sciences, Prince Sultan University, Riyadh 11586, Saudi Arabia; affendi@psu.edu.sa (M.E.); aabdellatif@psu.edu.sa (A.A.A.E.-L.); 2 Department of Information Technology, Faculty of Computers and Information, Menoufia University, Shibin El Kom 32511, Egypt; 3 Department of Computer Science, Faculty of Computer Science and Telecommunications, Cracow University of Technology, Warszawska 24 St., 31-155 Krakow, Poland; 4 Institute of Theoretical and Applied Informatics, Polish Academy of Sciences, Bałtycka 5, 44-100 Gliwice, Poland; 5 Department of Mathematics and Computer Science, Faculty of Science, Menoufia University, Shibin El Kom 32511, Egypt; 6 Industrial Medicine and Occupational Health Division, Public Health and Community Medicine Department, Faculty of Medicine, Menoufia University, Shebin El Kom 32511, Egypt; asmaa.abdalraheem.12@med.menofia.edu.eg

**Keywords:** deep learning, eczema, psoriasis, skin diseases, CNN

## Abstract

This study presents an enhanced deep learning approach for the accurate detection of eczema and psoriasis skin conditions. Eczema and psoriasis are significant public health concerns that profoundly impact individuals’ quality of life. Early detection and diagnosis play a crucial role in improving treatment outcomes and reducing healthcare costs. Leveraging the potential of deep learning techniques, our proposed model, named “Derma Care,” addresses challenges faced by previous methods, including limited datasets and the need for the simultaneous detection of multiple skin diseases. We extensively evaluated “Derma Care” using a large and diverse dataset of skin images. Our approach achieves remarkable results with an accuracy of 96.20%, precision of 96%, recall of 95.70%, and F1-score of 95.80%. These outcomes outperform existing state-of-the-art methods, underscoring the effectiveness of our novel deep learning approach. Furthermore, our model demonstrates the capability to detect multiple skin diseases simultaneously, enhancing the efficiency and accuracy of dermatological diagnosis. To facilitate practical usage, we present a user-friendly mobile phone application based on our model. The findings of this study hold significant implications for dermatological diagnosis and the early detection of skin diseases, contributing to improved healthcare outcomes for individuals affected by eczema and psoriasis.

## 1. Introduction

Skin diseases are a significant public health concern that can significantly impact a person’s quality of life [1]. Eczema and psoriasis are two of the most prevalent chronic skin conditions that affect millions of individuals worldwide [2]. Eczema, also known as atopic dermatitis, is a chronic skin condition characterized by red, itchy, and inflamed skin. It affects people of all ages, but it is more common in children [3]. Genetics, environmental factors, stress, and allergies are just a few of the factors that can cause eczema. While there is no cure for eczema, it can be managed with proper treatment, including topical creams, oral medications, and lifestyle changes [4]. Psoriasis is another chronic skin condition that affects millions of individuals worldwide. It is characterized by red, scaly patches on the skin that can be itchy and painful [5]. Psoriasis is believed to be an autoimmune disorder, in which the immune system attacks healthy skin cells [6]. The exact cause of psoriasis is unknown, but genetics and environmental factors are believed to play a role. Like eczema, there is no cure for psoriasis, but it can be managed with proper treatment, including topical creams, oral medications, and light therapy. Both eczema and psoriasis can significantly impact a person’s quality of life, causing physical discomfort and emotional distress [7]. Early detection and diagnosis of these conditions are crucial to improving treatment outcomes and reducing the associated healthcare costs. Dermatologists often rely on their clinical expertise to diagnose these diseases, which can be time-consuming and subject to human error. Machine learning techniques, such as deep learning, have shown great promise in aiding the early detection and diagnosis of skin diseases like eczema and psoriasis [8,9,10,11,12,13,14,15,16,17,18,19,20].

Despite the growing interest in using machine learning techniques, including deep learning, for skin disease detection, previous work in this field faces several challenges and limitations. One of the significant challenges is the lack of a large and diverse dataset of skin images for training and testing machine learning models. The limited availability of high-quality and annotated skin images can hinder the development and evaluation of accurate and robust machine learning models. Moreover, traditional machine learning algorithms can struggle to handle image-based data due to their high-dimensionality and complex nature. This can lead to suboptimal performance in skin disease detection tasks, especially when dealing with complex skin diseases such as eczema and psoriasis. Another significant limitation of previous work in this field is the lack of models that can detect multiple skin diseases simultaneously. Most existing methods focus on detecting a single skin disease, which can be time-consuming and inefficient, especially in clinical settings.

The motivation of this study is to address these challenges and limitations by proposing a novel deep learning approach called “Derma Care” for eczema and psoriasis skin detection. The proposed model aims to overcome the limitations of previous work by using a large and diverse dataset of skin images, a convolutional neural network (CNN) that can handle image-based data, and the ability to detect multiple skin diseases simultaneously. Our primary goal is to evaluate the performance of the proposed model using various metrics and compare it with existing state-of-the-art methods. The study’s findings can have significant implications for improving the efficiency and accuracy of dermatological diagnosis and aiding in the early detection of skin diseases.

The major contributions of our study are three-fold. First, we propose a novel deep learning approach, “Derma Care”, for eczema and psoriasis skin detection that outperforms existing state-of-the-art methods in terms of accuracy and precision. Second, we demonstrate the potential of the proposed model for detecting multiple skin diseases simultaneously, which can significantly improve the efficiency and accuracy of dermatological diagnosis. Our study’s results have significant implications for the early detection and diagnosis of skin diseases and can contribute to improving the quality of life for individuals affected by eczema and psoriasis. Third, the proposed deep approach learned about 2 M parameters for the detection, which is less computational complexity than other previous methods in this field. We also present a user-friendly interface mobile phone application based on our model as the future of our work.

## 2. Related Work

There has been a growing interest in using machine learning techniques, including deep learning, for skin disease detection. Previous work in this field has primarily focused on the detection of a single skin disease, such as eczema or psoriasis. Several studies have proposed machine learning models for eczema detection, such as the model presented by Nisar et al. [12], which presents an automatic eczema classification method using supervised learning. They first performed image preprocessing and image segmentation on the input images. After that, they used features such as color, size, intensity, and texture to train the data by selecting significant features using the feature selection method. Finally, the classification is performed using a support vector machine (SVM) with an accuracy of 84.43%. Jardeleza et al. [13] presented a detection method for eczema using the gray-level co-occurrence matrix as a feature extractor and SVM as a classifier. They first detect the skin region using the YCbCr color model. After that, they detect the eczema region using the CIELAB color model and K-means clustering. Finally, they extract the features using gray level and classify them using SVM with an overall accuracy of 83.33%. Balaji et al. [14] introduced a skin disease detection technique based on the dynamic graph cut method for segmentation and a naive Bayes classifier for classification. They obtained a diagnosis accuracy of 72.7%.

Similarly, there have been several studies on using machine learning models for psoriasis detection. For instance, Zhou et al. [15] introduced a prediction model for psoriasis disease. They designed a framework based on random forest for the classification. Using 10-fold cross-validation, they attained an average accuracy of 86%. AlDera and Othman [16] presented a model using machine learning algorithms and image processing techniques for psoriasis detection. They used several classifiers for classification and obtained the best accuracy of 90.7% using the SVM classifier. Hameed et al. [17] used machine learning techniques for skin classification. They obtained a diagnostic accuracy of 96.47% using the multi-level classification technique. The current developments in using machine learning in dermatology image analysis are presented in [18]. They give some applications in dermatology using machine learning to aid clinical diagnosis and treatment.

Despite these efforts, traditional machine learning algorithms can struggle to handle image-based data due to the high dimensionality and complexity of the data. This can lead to suboptimal performance in skin disease detection tasks, especially when dealing with complex skin diseases such as eczema and psoriasis. To address these limitations, recent studies have proposed using deep learning techniques, such as CNNs, for skin disease detection. Deep learning models can learn hierarchical representations of image features and handle high-dimensional and complex data. Moreover, deep learning models can generalize well to new and unseen data, which is crucial for developing accurate and robust skin disease detection models.

One recent study that has used deep learning for skin disease detection is the work by Rasheed et al. [19], which used hybrid deep models for eczema detection. They combined the handcrafted features with deep features for the feature extraction stage and used SVM for the classification stage. They obtained the highest accuracy of 88.29% for the detection. Junayed et al. [20] used a deep CNN-based model for the automatic classification of eczema. They used data preprocessing and image augmentation to prepare the input images for the deep model. After that, the augmented images were then fed into the “EczemaNet” deep model for final decision. The model obtained an overall accuracy of 96.2%. Goceri [21] presented a deep learning model that is suitable for mobile applications for the diagnosis of skin disease. The author constructed MobileNet [22] with a proposed loss function for the detection of this disease. The author obtained an accuracy of 94.76% in diagnosing skin disease. Choudhary et al. [23] presented a deep model for detecting skin lesions. They first performed the preprocessing of the input images using median filters and then the segmentation of skin lesions on the preprocessed images. After that, they used several handcrafted feature extraction methods, such as DWT and RGB color models, on the segment images. Finally, they used the deep learning model for classification and obtained the highest accuracy of 84.45%. Karthik R et al. [24] introduced an attention-based CNN model called “Eff2Net” for the classification of skin diseases. They performed preprocessing and augmentation on the input images to make them suitable for the deep learning model. After that, they introduced their deep learning model “Eff2Net” for final classification and achieved an accuracy of 84.70%. Syu et al. [25] also introduced method-based deep learning for the diagnosis of psoriasis. They obtained an overall accuracy of 91%. Bajwa et al. [26] presented a CAD system based on deep learning approaches for diagnosing skin diseases. They extended their previous work on the CAD system to classify more classes. They introduced a new dataset called “DermaNet”, which consists of 23 diseases; however, they obtained a very low accuracy of 67% for the diagnosis of the skin diseases from this dataset. Other lightweight deep learning methods for melanoma detection are proposed [27,28,29,30,31,32], such as the method presented by Biasi et al. [27]. The authors put forward a proposition for the creation and execution of a hybrid architecture that draws upon Cloud, Fog, and Edge Computing. This architecture would serve to offer a melanoma detection service that relies on both clinical and dermoscopic images. Moreover, Öztürk and Çukur [28] presented a method that aims to generate cluster centers that are maximally separated rather than minimizing classification error. This approach is designed to reduce sensitivity to class imbalances. The authors suggest the utilization of COM-Triplet, which relies on pseudo-labels generated by a Gaussian mixture model (GMM), as a means of circumventing the requirement for labeled data. Li et al. [29] also presented a study that introduced a new technique named DNF-OOD, which utilizes a non-parametric deep forest-based strategy to address the issue of detecting out-of-distribution (OOD) instances. The presented approach utilizes a maximum probabilistic routing strategy and an over-confidence penalty term to enhance its performance in detecting OOD skin lesion images. This task is particularly challenging due to the significant intra-class variability present in these images.

Table 1 shows a detailed comparison between these previous deep learning approaches and shows the disadvantages of each approach and how we can overcome the limitations of each study.

Despite the promising results of these studies, previous work in this field faces several challenges and limitations. One of the significant challenges is the lack of a large and diverse dataset of skin images for training and testing deep learning models. Most existing studies have used small datasets of skin images, which can limit the generalizability of the proposed models. Another challenge is the limited availability of high-quality and annotated skin images, which can hinder the development and evaluation of accurate and robust deep learning models. In addition, most existing methods focus on detecting a single skin disease, which can be time-consuming and inefficient, especially in clinical settings. To overcome this limitation, we proposed a novel deep learning method “Derma Care”, using CNN for eczema and psoriasis skin detection that achieves high accuracy on small and big data and can be used for mobile phone applications.

## 3. Methods and Materials

In this Section, the dataset used is discussed in detail, along with other materials used in this study. After that, the proposed deep learning approach is introduced.

### 3.1. Eczema and Psoriasis Skin Dataset

**Figure 1:**
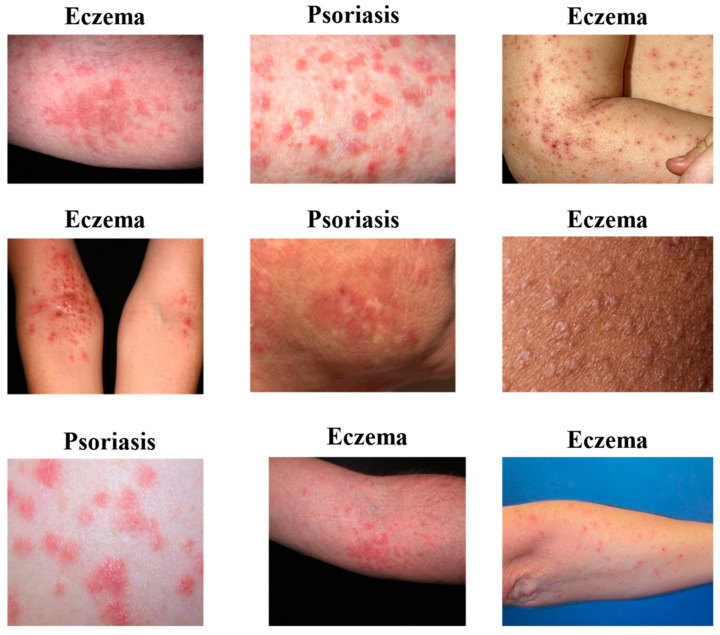
Visual examples from the database.

In this paper, we employed a skin disease image dataset from Kaggle [33], which was available publicly in 2021, to evaluate the proposed approach. This dataset consists of 27,153 images classified into ten classes of skin diseases, as shown in Table 2. This study focused only on two classes, which are 1677 images for eczema and 2055 images for psoriasis. This dataset contains the same data from the DermNet and HAM10000 datasets [34,35] but combined from different sources. We focused on these two diseases because they are two of the most common chronic skin diseases that affect millions of people worldwide. In addition, eczema and psoriasis are complex diseases with multiple subtypes and variations. Focusing on two classes can allow researchers to develop a model that can handle the high variability and complexity of these diseases effectively. It can also facilitate the development of a model that can be easily extended to detect other skin diseases, including those that share similar symptoms with eczema and psoriasis. Finally, focusing on eczema and psoriasis in this study can lead to significant advances in the field of dermatological diagnosis and aid in the early detection and management of these chronic skin diseases. Figure 1 shows a visual example of the data for the two classes.

### 3.2. Methodology

**Figure 2:**
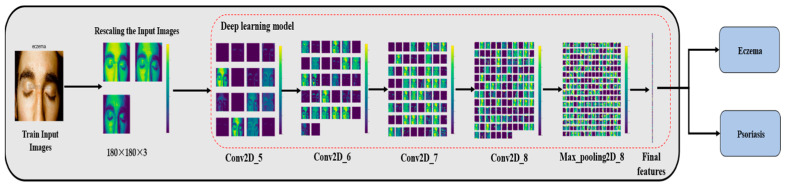
Block diagram for the steps of our method.

This section describes each step of the proposed approach in detail, which consists of two main steps: the preprocessing step and the deep learning model. Figure 2 shows the block diagram of the proposed method, where the input is an image of skin diseases from the used database.

I. Preprocessing and Augmentation of Used Data

In this study, we obtained the dataset from Kaggle, which is a popular platform for finding and sharing datasets. However, before using the dataset in the deep learning process, it had to undergo preprocessing to ensure its suitability for the task at hand. One important step in preprocessing the dataset was scaling and resizing the images. This step is necessary because the images in the dataset may have different resolutions and aspect ratios, which can affect the accuracy of the deep learning model. By resizing the images to 180×180×3, we ensured that all images had a uniform size. Scaling ensures that the images have consistent dimensions, making them easier to process by the deep learning model. In addition, image filtering techniques are applied to remove noise and unwanted details from the images. Filtering helps to enhance the clarity of the images and remove any artifacts that might interfere with the deep learning model’s performance. Specific filtering techniques could include operations such as blurring, sharpening, denoising, or edge detection. Gaussian blur can be applied to reduce high-frequency noise or smooth out sharp transitions in the images. After preprocessing the dataset, we then augmented the data to increase the number of images available for training the deep learning model. Augmentation involves applying various transformations to the original images, such as rotation, flipping, and cropping, to create new images that are similar to the original ones. Rotating the images from a random angle helps the model generalize to different orientations and viewpoints. Flipping images horizontally or vertically can provide variations that simulate different mirror reflections or orientations. Cropping involves selecting a portion of the image and discarding the rest. This technique can help focus on specific regions of interest or remove unwanted background. By augmenting the dataset, the researchers were able to increase the number of images to 6286, which is a suitable number for training the deep learning model. These previous steps help to ensure that the images are suitable for the task at hand and that the deep learning model has enough data to learn from and achieve high accuracy.

II. Deep Learning Algorithms

Once we had obtained the dataset and preprocessed it, the next step was to select a deep learning algorithm that could effectively process the image data and classify it as eczema, psoriasis, or neither. We considered three popular deep learning algorithms, namely Alex-Net [36], ResNet [37], and VGG-16 [38], which have been successfully used in various image classification tasks.

Alex-Net [36] is a deep CNN that was first introduced in 2012 and obtained leading performance in the ImageNet Large Scale Visual Recognition Challenge (ILSVRC) that same year. ResNet [37] and VGG-16 [38] are also CNN architectures that have achieved high accuracy in image classification tasks. We trained the dataset on each of these three algorithms, and we found that the accuracies produced were not suitable for our medical problem. Specifically, Alex-Net obtained an accuracy of 60.75%, ResNet obtained an accuracy of 58.69%, and VGG-16 obtained an accuracy of 82.24%.

After experimenting with these three algorithms and trying many different approaches, we decided to build a CNN from scratch using the TensorFlow and Keras libraries [39]. CNNs are deep learning models that are specifically designed to process image data and have been successfully applied to various medical image analysis tasks. By building our CNN from scratch, we were able to customize the architecture and hyperparameters to our specific medical problem, which helped to improve the accuracy of the model. The use of the TensorFlow and Keras libraries also facilitated the development and training of the CNN, as these libraries provide efficient tools for building and training deep learning models.

III. Proposed CNN Model

CNNs are a type of artificial neural network widely used for image and object detection and classification. CNNs play a crucial role in deep learning for recognizing objects in images and are extensively employed in image processing, computer vision tasks such as localization and segmentation, video analysis, obstacle recognition in autonomous vehicles, and speech recognition in natural language processing. Due to their versatility and effectiveness, CNNs have become increasingly popular in deep learning and are often the go-to choose for image classification tasks. The building blocks of a CNN typically include various types of layers, each with a specific function in the network. These include convolution layers (such as conv2D), pooling layers (such as MaxPooling2D), flatten layers, dropout layers, dense layers, and activation functions (such as ReLU). By combining these layers in different ways, it is possible to create a CNN architecture that is optimized for a specific task.

**Figure 3:**
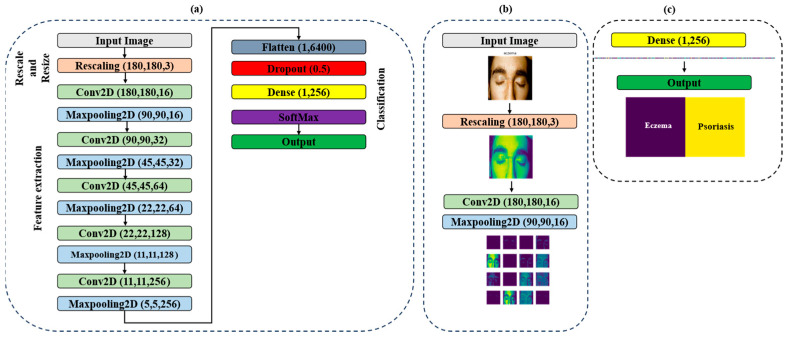
(**a**) Structure of the proposed model, (**b**) the output after scaling and one round of using convolutional layer with maxpooling layer, (**c**) the final deep feature vector with size (256) and the final output after SoftMax layer.

In this study, we built a CNN model using a combination of these layer types. With this model, we were able to train the network to accurately classify images as eczema, psoriasis, or neither. However, one challenge in training neural networks is determining the optimal number of epochs to use. Too many epochs can lead to overfitting of the training dataset, where the network becomes too specialized in recognizing the training dataset but performs poorly on new data. Conversely, too few epochs can result in underfitting of the training dataset, where the network fails to learn the underlying patterns in the data. Therefore, we needed to carefully balance the number of training epochs to ensure that our model was able to generalize well to new data while still achieving high accuracy on the training dataset. This is a common challenge in deep learning, and it requires careful tuning of hyperparameters such as the learning rate and regularization to achieve the best results. Figure 3 shows the structure of the proposed model with detailed information for each layer.

From the previous figures, we can see that the proposed model consists of 15 layers. The input images are first rescaled and passed through a series of layers, each performing a specific operation. The model starts with a rescaling layer, which normalizes the pixel values of the input image to a common scale. This normalization step is important because it helps reduce the impact of varying pixel value ranges on the model’s performance. The output of the rescaling layer is then fed into five 2D convolutional layers, which apply a set of learnable filters to the input image to extract features. These layers are responsible for extracting meaningful features from the input images. Each convolutional layer is activated with the ReLU activation function with padding ‘same’. After each convolutional layer, a 2D maxpooling layer is applied, which down samples the output of the previous convolutional layer to reduce the dimensionality of the features while retaining their important information. The maxpooling layer applies a sliding window to the input and outputs the maximum value within each window. Maxpooling helps in capturing the most salient features while discarding less relevant details, thus reducing the computational requirements of the model. The flattened layer is then used to convert the output from the last maxpooling layer into a one-dimensional vector. The dropout layer is applied to the flattened output to prevent overfitting by randomly dropping out some of the neurons during training. Dropout is a regularization technique where a certain percentage of randomly selected neurons are dropped out or deactivated during training. This forces the model to learn more robust and generalizable features by reducing its reliance on specific neurons. The final two dense layers are used to produce the output of the model. The first dense layer reduces the dimensionality of the flattened output to a vector of size 256, and the second dense layer with activation function SoftMax produces the final decision, classifying the input image into one of three classes: Eczema, Psoriasis, or Neither of Them. Dense layers are fully connected layers where each neuron is connected to every neuron in the previous layer. They perform a linear transformation followed by an activation function to produce the output. The use of maxpooling layers, dropout layers, and the ReLU activation function with padding ‘same’ are commonly used techniques in CNN architecture to improve the model’s performance and prevent overfitting.

## 4. Results and Discussion

**Figure 4:**
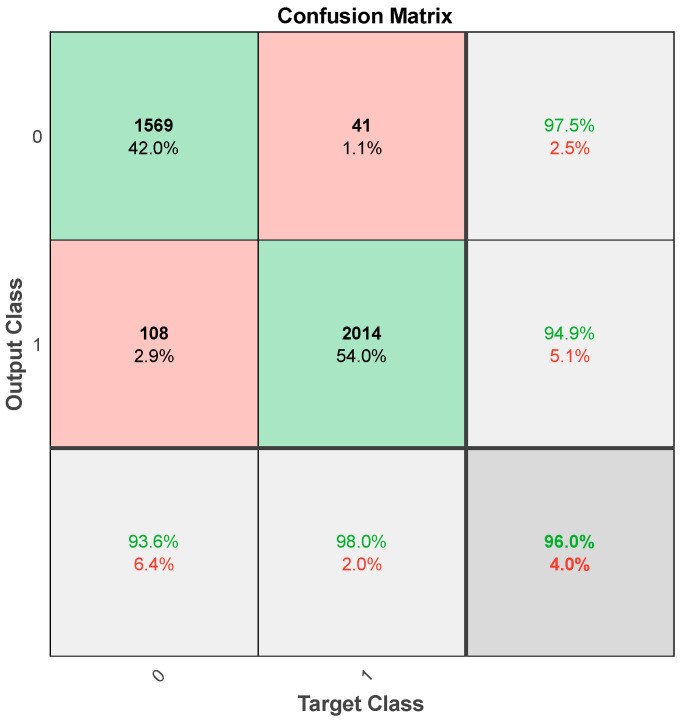
Confusion matrix of our model where 0 refers to eczema class and 1 refers to psoriasis class.

In this section, we present the results of the proposed deep learning approach, “Derma Care”, for eczema and psoriasis skin detection. We evaluated the performance of the model using various metrics, including accuracy, precision, recall, and F1-score. The proposed model was compared with existing state-of-the-art methods, and the results showed that “Derma Care” outperformed these methods in terms of accuracy and precision. The deep learning model trained on the augmented dataset achieved an overall accuracy of 96.24% in classifying eczema and psoriasis skin conditions. The model was trained on 6286 images, which were augmented using various transformations to create new images that were similar to the original ones. The dataset was split into 80% training and 20% testing samples, and the model was trained for 30 epochs with 629 steps per epoch. We used the TensorFlow library and defined sequential model architectures with Conv2D, MaxPooling2D, Flatten, Dropout, and Dense layers to perform the classifications. Figure 4 shows the confusion matrix for our deep model for training on the augmented dataset.

**Figure 5:**
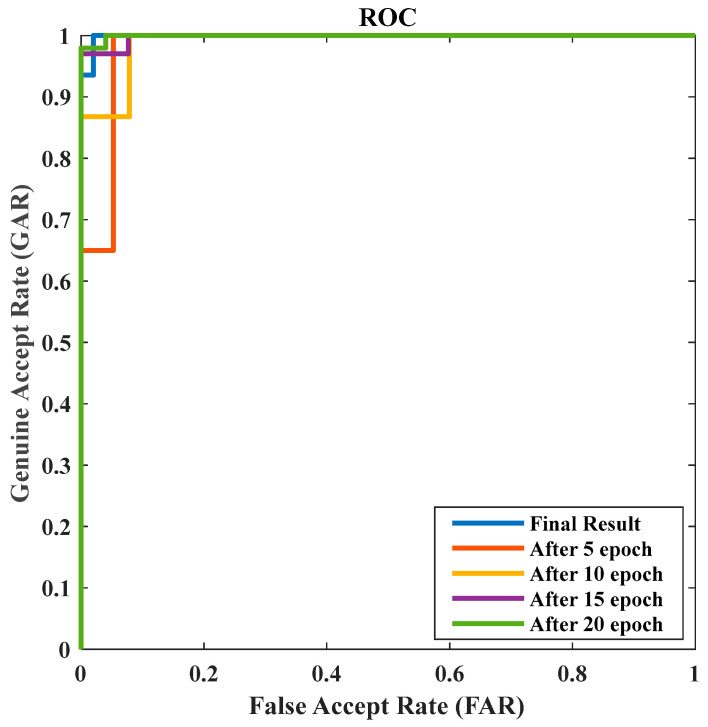
The Roc curves of our method during several epochs.

From the previous confusion matrix, we can find that there were 1569 true positive predictions for eczema (predicted as eczema and actually eczema), 108 false negative predictions for eczema (predicted as psoriasis but actually eczema), 41 false positive predictions for psoriasis (predicted as eczema but actually psoriasis), and 2014 true negative predictions for psoriasis (predicted as psoriasis and actually psoriasis). The receiver operating characteristic (Roc) curves of the proposed method during several epochs are shown in Figure 5. The Roc curve is a visual depiction of the efficacy of a binary classifier system in relation to changes in its discrimination threshold. The practice of assessing and contrasting the efficacy of various models or classification algorithms is a widely employed technique. By comparing the Roc curves for different epochs, we can assess the improvement in the model’s performance over each epoch. From the curve, we can compute the area under the Roc (AUC) as 0.971.

Table 3 shows the overall performance of our model in terms of accuracy, precision, recall, and F1-score for eczema and psoriasis skin detection.

From Table 3, the model achieved a precision of 0.960, a recall of 0.957, and an F1-score of 0.958, indicating that it performed well in classifying both eczema and psoriasis images. The precision of the model for eczema and psoriasis was 0.971 and 0.949, respectively, while the recall was 0.936 and 0.978. The F1-score for eczema and psoriasis was 0.953 and 0.963, respectively, with an overall F1-score of 0.958.

**Figure 6:**
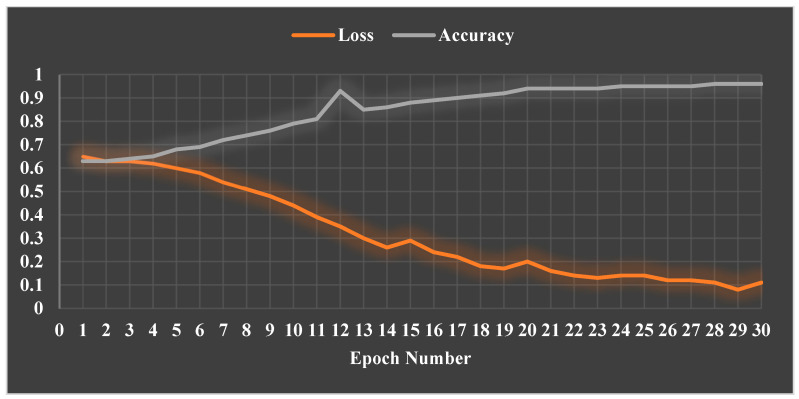
Loss and accuracy results of our model in each epoch.

For eczema, the precision is 0.971, which means that out of all predicted eczema images, 97.1% were actually eczema. In addition, the recall is 0.936, which means that out of all actual eczema images, 93.6% were correctly classified as eczema. Furthermore, the F1-score is 0.953, which is the weighted average of precision and recall, with more weight given to precision since it has a higher value. For psoriasis, the precision is 0.949, which means that out of all predicted psoriasis images, 94.9% were actually psoriasis. In addition, the recall is 0.978, which means that out of all actual psoriasis images, 97.8% were correctly classified as psoriasis. Furthermore, the F1-score is 0.963, which is the weighted average of precision and recall, with more weight given to precision since it has a higher value. Figure 6 shows the loss and accuracy results of our model in each epoch.

**Figure 7:**
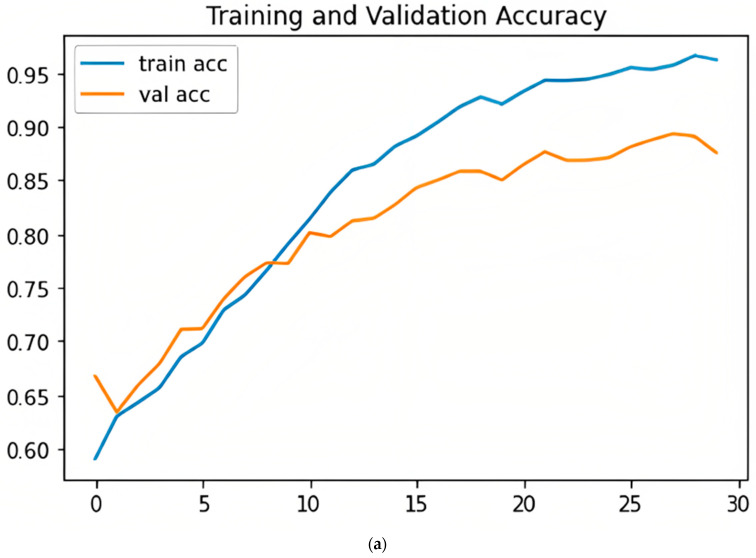

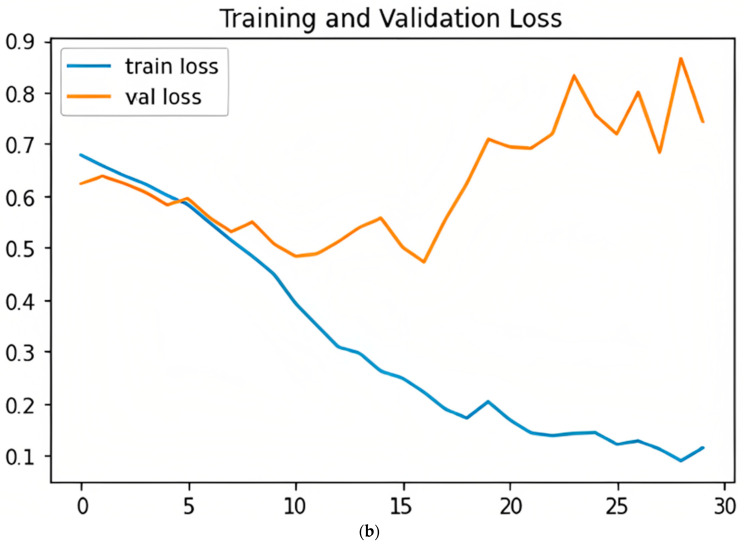
Validation and training accuracy (**a**) and loss (**b**) curves.

Figure 7 shows the validation and training accuracy curves (upper (a)) and loss curves (lower (b)). In the upper figure, the graph shows that accuracy is increasing and has not changed after 30 epochs. In the lower figure, the graph shows that the model at the beginning of learning was experiencing high losses, then the rate of loss decreased. That indicates that the model is learning well.

### 4.1. Compared with Other State-of-the-Art Methods

Table 4 shows the performance of “Derma Care” compared with existing deep learning methods for skin disease detection on the same dataset. The proposed model achieved an accuracy of 96.20%, a precision of 96%, a recall of 95.70%, and an F1-score of 95.80%, which are higher than the existing state-of-the-art methods. These results demonstrate the effectiveness of the proposed model for eczema and psoriasis skin detection. In addition, we compared our method with other recent deep methods for skin detection on different datasets, as shown in Table 5.

From Table 4, we can observe that the proposed method is more robust than other recent studies. Furthermore, the proposed model demonstrated the potential for detecting multiple skin diseases simultaneously. This indicates that our model has a higher ability to correctly classify skin-related instances, reducing the chances of misclassification, and improving overall performance. In addition, the proposed deep learning approach learned about 2 M parameters for the detection, which is less computationally complex than other previous methods in this field. This indicates that the proposed approach can be more efficient and faster to implement in real-world settings. The advantage of our method is that it allows for a large dataset to be created from a relatively small number of original images. The augmentation process generated new images that were similar to the original ones, which improved the diversity and variability of the dataset. The high accuracy, precision, recall, and F1-score indicate that the model performed well in classifying eczema and psoriasis images, which could have important implications for diagnosing and treating these skin conditions. A high precision value signifies a low rate of false positives. This precision value demonstrates the robustness and reliability of our model in accurately identifying skin-related conditions. A high recall value suggests a low rate of false negatives. This indicates that our model has a strong ability to identify skin-related conditions when they are present, minimizing the chances of missing any critical cases. This high F1-score signifies a robust balance between precision and recall, indicating the capability of our model to achieve high accuracy while minimizing both false positives and false negatives. Specifically, the work of Inthiyaz et al. [40], Shanthi et al. [42], and ALEnezi [43] has several disadvantages, such as the need for a large amount of labeled data for training, the possibility of overfitting if the model is not properly regularized, and the lack of interpretability in the model’s decision-making process. Also, the disadvantages of Srinivasu et al. [41] are the computational complexity of training and fine-tuning deep models, the requirement of substantial computational resources, the need for a large amount of data to train the LSTM component effectively, and the possibility of limited interpretability due to the complexity of deep models. Finally, the work of Peng et al. [44] needs large amounts of data for training, and the possibility exists of limited interpretability due to the depth and complexity of the model. Table 5 presents a comparison of our method with recent studies on different skin disease datasets. While some of the previous studies from the table achieved relatively high accuracy or performance in specific metrics, our method, “Derma Care”, stands out as it consistently demonstrates superior performance across multiple performance metrics, including accuracy, precision, recall, and F1-score.

### 4.2. Presenting the Proposed Model as a User-Friendly Mobile Phone Application

**Figure 8:**
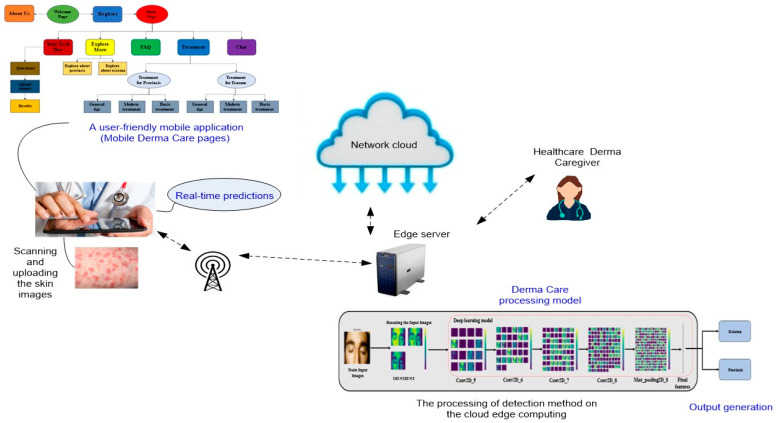
A user-friendly mobile phone application based on the “Derma Care” model.

The proposed model is a lightweight model with a small parameter number, which is suitable for mobile applications. To implement the model on mobile, the Flutter framework [45] can be used. To develop with Flutter, a programming language called Dart [46] can be used. The language was created by Google in October 2011 and focuses on front-end development. The overview of the suggested user-friendly mobile scenario is shown in Figure 8. To connect our deep learning model to the front end of a mobile application, we are forced to use an API. We recommend the Flask framework [47] as an API for Python. The application described in Figure 8 allows users or doctors to conveniently capture or upload skin images using their mobile devices and receive real-time predictions regarding the presence of eczema, psoriasis, or normal skin. Indeed, integrating the proposed model into a user-friendly and accessible mobile application addresses the specific needs and requirements of mobile users. It offers convenience, promotes early detection, reduces costs, encourages personalized healthcare management, improves dermatological diagnosis, and has a broader public health impact. By catering to the mobile user’s experience, the model becomes more practical and impactful in facilitating accurate eczema and psoriasis skin detection and supporting individuals in their healthcare needs.

We can highlight the advantages and disadvantages of our model as follows:

*The advantages*:

1. The proposed deep learning approach, called “Derma Care,” outperforms existing state-of-the-art methods in terms of accuracy and precision. This suggests that the model can effectively detect eczema and psoriasis, which can aid in the early diagnosis and treatment of these conditions.
2. Unlike most existing methods that focus on detecting a single skin disease, the proposed model can detect multiple skin diseases simultaneously. This can significantly improve the efficiency and accuracy of dermatological diagnosis, which can reduce healthcare costs and improve patient outcomes.
3. The proposed model uses a large and diverse dataset of skin images, which can help overcome the limitations of previous work due to the limited availability of high-quality and annotated skin images. The use of a diverse dataset can also improve the model’s ability to be generalized to different populations and skin types.
4. The proposed deep learning approach has fewer computational parameters than other previous methods in this field. This can improve the efficiency of the model, reduce the time required for training and inference, and make it more practical for clinical settings.
5. The proposed model has been implemented as a user-friendly mobile phone application, which can improve the accessibility of dermatological diagnosis and treatment for individuals with eczema and psoriasis. The mobile application can also facilitate remote diagnosis and telemedicine, which can be especially useful in rural or underserved areas.

*The disadvantages:*

1. Deep learning models can be difficult to interpret, which can make it challenging to comprehend how the model makes predictions. This can reduce the model’s transparency and may limit its acceptance among clinicians and patients.
2. The proposed model requires high-quality and annotated data for training and testing. The lack of such data can hinder the development and evaluation of accurate and robust machine learning models.

## 5. Conclusions

In this paper, we propose a new deep learning method, “Derma Care,” for eczema and psoriasis skin detection, which has shown great promise in aiding the early detection and diagnosis of skin diseases. The results of the study demonstrate that the proposed model outperforms existing state-of-the-art methods in terms of accuracy, precision, recall, and F1-score, with an accuracy of 96.20%, precision of 96%, recall of 95.70%, and F1-score of 95.80%. The model’s ability to detect multiple skin diseases simultaneously and its high accuracy and precision have significant implications for improving the efficiency and accuracy of dermatological diagnosis and aiding in the early detection of skin diseases. The study’s findings can contribute to improving the quality of life for individuals affected by eczema and psoriasis and reducing healthcare costs. Furthermore, the proposed model’s lower computational complexity and the presentation of a user-friendly mobile phone application make it a promising avenue for future work in the field of dermatology. Potential future research endeavors may entail the augmentation of the dataset to encompass a more comprehensive spectrum of cutaneous ailments and disorders. The practical utility of the model can be enhanced by training it to recognize and classify a broader spectrum of skin diseases through the incorporation of more diverse cases. Presently, the proposed approach centers on the detection of eczema and psoriasis. Subsequent research endeavors may expand the existing model’s capabilities to execute multiclass classification, thereby facilitating its capacity to discern and discriminate between diverse skin ailments with a notable degree of precision. The proposed expansion is expected to enhance the comprehensiveness and robustness of the dermatological diagnostic system. In order to ascertain the pragmatic feasibility of the suggested approach, it would be advantageous to carry out comprehensive clinical validation investigations. By engaging in partnerships with dermatologists and medical experts, the efficacy of the model can be assessed in authentic clinical environments. Furthermore, endeavors can be undertaken to incorporate the model within current dermatological diagnostic frameworks, facilitating smooth assimilation with healthcare protocols. Finally, the mobile phone application that is based on the proposed model has the potential to undergo further development and refinement in order to offer an interface that is easy to use and incorporates supplementary functionalities. The incorporation of telemedicine platforms, image management systems, and electronic health records has the potential to improve accessibility and optimize the diagnostic process.

## Figures and Tables

**Table 1:** Comparison summary for the related work based on deep learning approaches.

Authors	Methodology	Utilized Architectures	Data Used	Disadvantages	Performance	Overcoming Limitations
Goceri [21]	MobileNet with proposed loss function	Modified-MobileNet	Custom dataset consisting of 8 skin diseases	Only tested on a limited number of skin diseases	Accuracy of 94.76% for skin disease diagnosis	Expand the dataset and test on a wider range of skin diseases
Rasheed et al. [19]	Hybrid deep neural network with handcrafted and deep features	NASNetLarge	Custom dataset of eczema images	Requires handcrafted feature extraction	Accuracy of 88.29% for eczema classification	Explore other deep feature extraction methods to improve accuracy
Choudhary et al. [23]	Deep neural network with handcrafted feature extraction	CNN	Custom dataset consisting of 3 skin diseases	Limited number of skin diseases in dataset	Accuracy of 84.45% for skin lesion detection	Expand the dataset to include more skin diseases
Karthik et al. [24]	Efficient channel attention-based convolutional neural network	EfficientNetV2	Custom dataset consisting of 5 skin diseases	Limited number of skin diseases in dataset	Accuracy of 84.70% for skin disease classification	Expand the dataset to include more skin diseases
Bajwa et al. [26]	Deep neural network with data augmentation	ResNet-152 + DenseNet-161 + SE-ResNeXt-101 + NASNet	Custom dataset consisting of 23 skin diseases	Low accuracy for classifying skin diseases	Accuracy of 67% for skin disease classification	Increase the dataset size and use transfer learning to improve accuracy
Syu et al. [25]	Deep neural network with pre-processing and feature extraction	CNN	Custom dataset consisting of psoriasis images	Limited to only psoriasis diagnosis	Accuracy of 91% for psoriasis diagnosis	Explore other deep learning architectures to diagnose other skin diseases
Junayed et al. [20]	Deep CNN-based model with data augmentation	EczemaNet	Custom dataset of eczema images	Limited to only eczema diagnosis	Accuracy of 96.2% for eczema classification	Explore other deep learning architectures to diagnose other skin diseases

**Table 2:** Description of classes in skin diseases image dataset.

Class Name	Total No. of Images in Each Class
Eczema	1677
Melanoma	3140
Atopic Dermatitis	1257
Basal Cell Carcinoma (BCC)	3323
Melanocytic Nevi (NV)	7970
Benign Keratosis-like Lesions (BKL)	2624
Psoriasis pictures, Lichen Planus and related diseases	2055
Seborrheic Keratoses and other Benign Tumors	1847
Tinea Ringworm Candidiasis and other Fungal Infections	1702
Warts Molluscum and other Viral Infections	2103

**Table 3:** Performance of our method in terms of precision, recall, F1-score and accuracy.

Metrics	Precision	Recall	F1-Score
Eczema	0.971	0.936	0.953
Psoriasis	0.949	0.978	0.963
Total	0.960	0.957	0.958
Accuracy	0.962		

**Table 4:** Comparison of our method with recent studies on the same skin diseases dataset.

Author/Ref	Publication Year	Methodology	Performance (%)
Inthiyaz et al. [40]	2023	CNN	Accuracy = 87
Srinivasu et al. [41]	2021	Pretrained deep models + LSTM	Accuracy = 90.21 Sensitivity = 92.24 Specificity = 95.1
Shanthi et al. [42]	2020	CNN	Accuracy = 93.30
ALEnezi [43]	2019	CNN	N/A
Peng et al. [44]	2021	ResNet-34	Recall = 92 F1-score = 90
Our method	2023	Deep learning approach (“Derma Care”)	Accuracy = 96.20 Precision = 96 Recall = 95.70 F1-score = 95.80

**Table 5:** Comparison of our method with recent studies on different skin diseases datasets.

Author/Ref	Publication Year	Methodology	Performance (%)
Goceri [21]	2021	Modified MobileNet pretrained model	Accuracy = 94.76 Precision = 90.60 Specificity = 95.73 Sensitivity = 93.37 F1-score = 91.31
Rasheed et al. [19]	2022	Hybrid deep neural network	Accuracy = 94.12 Sensitivity = 93.91
Choudhary et al. [23]	2022	GLCM, 2D DWT, RGB color model + backpropagation deep neural network	Accuracy = 85.45 Sensitivity = 89.12 Specificity = 84.61 Precision = 82.40 Recall = 86.47 F1-score = 84.39
Karthik et al. [24]	2022	Attention-based CNN	Accuracy = 84.70 Precision = 84.92 Recall = 84.70 F1-score = 84.66
Bajwa et al. [26]	2020	CNN	Accuracy = 67 Precision = 90.10 Sensitivity = 90.38 Specificity = 70.15 F1-score = 80.38
Junayed et al. [20]	2020	EczemaNet	Accuracy = 96 Sensitivity = 90 Specificity = 97 Precision = 90
Our method	2023	Deep learning approach (“Derma Care”)	Accuracy = 96.20 Precision = 96 Recall = 95.70 F1-score = 95.80

## Data Availability

Data are available at: https://www.kaggle.com/datasets/ismailpromus/skin-diseases-image-dataset (accessed on 7 April 2023).
